# Improving the efficiency of genomic loci capture using oligonucleotide arrays for high throughput resequencing

**DOI:** 10.1186/1471-2164-10-646

**Published:** 2009-12-31

**Authors:** Hane Lee, Brian D O'Connor, Barry Merriman, Vincent A Funari, Nils Homer, Zugen Chen, Daniel H Cohn, Stanley F Nelson

**Affiliations:** 1 Department of Human Genetics, University of California, Los Angeles, California, USA; 2 Medical Genetics Institute, Cedars-Sinai Medical Center, Los Angeles, California, USA

## Abstract

**Background:**

The emergence of next-generation sequencing technology presents tremendous opportunities to accelerate the discovery of rare variants or mutations that underlie human genetic disorders. Although the complete sequencing of the affected individuals' genomes would be the most powerful approach to finding such variants, the cost of such efforts make it impractical for routine use in disease gene research. In cases where candidate genes or loci can be defined by linkage, association, or phenotypic studies, the practical sequencing target can be made much smaller than the whole genome, and it becomes critical to have capture methods that can be used to purify the desired portion of the genome for shotgun short-read sequencing without biasing allelic representation or coverage. One major approach is array-based capture which relies on the ability to create a custom in-situ synthesized oligonucleotide microarray for use as a collection of hybridization capture probes. This approach is being used by our group and others routinely and we are continuing to improve its performance.

**Results:**

Here, we provide a complete protocol optimized for large aggregate sequence intervals and demonstrate its utility with the capture of all predicted amino acid coding sequence from 3,038 human genes using 241,700 60-mer oligonucleotides. Further, we demonstrate two techniques by which the efficiency of the capture can be increased: by introducing a step to block cross hybridization mediated by common adapter sequences used in sequencing library construction, and by repeating the hybridization capture step. These improvements can boost the targeting efficiency to the point where over 85% of the mapped sequence reads fall within 100 bases of the targeted regions.

**Conclusions:**

The complete protocol introduced in this paper enables researchers to perform practical capture experiments, and includes two novel methods for increasing the targeting efficiency. Coupled with the new massively parallel sequencing technologies, this provides a powerful approach to identifying disease-causing genetic variants that can be localized within the genome by traditional methods.

## Background

Sequencing capacity has greatly advanced over the years and took a major leap with the commercialization of two new platforms for next-generation sequencers. Currently, three major platforms are actively being used including Applied Biosystems (ABI) SOLiD (Sequencing by Oligo Ligation and Detection), Illumina Genome Analyzer (GA) and Roche 454 Sequencing System [1-3]. Further, proofs of principle experiments with the Helicos system and Pacific Biosciences system have been published [4-6]. These sequencer technologies differ in their sequencing methods and hence they vary in number of reads sequenced, read length, error characteristics. However, all rely on the generation of shotgun libraries for sequencing. With these technologies, a single machine can generate in the range of 0.5-2 gigabases (Gb) of sequence reads per day. While advancement in these technologies is certain, the use of these technologies for sequencing targeted regions of the genome has been limited based on the efficiency of methods to enrich regions of the genome for analysis which are matched to the capacity. The rapid advancement in genotyping technology made possible by the advent of DNA microarrays has resulted in a flood of linkage and whole genome association studies for various disorders, and now the community is overwhelmed with genomic regions of interest for which additional targeted sequence analysis is key bottleneck. Most recently, several studies on exonic capture for broad based sequencing of the amino acid coding portion of the genome have shown successful identification of rare mutations/alleles involved in rare genetic disorders and yielded insights in applying the technique to searching for common variants as well as *de novo *cancer mutations [7-10].

Several groups have attempted to capture regions of interest by multiplex amplification [11-14]. For the target regions, primer pairs are systematically designed and, as the target regions are amplified by PCR, only the fragments with the right primer pairs are enriched. Reports have demonstrated successful amplification of hundreds of ~200 bp sized fragments but with substantial bias in the amplification between the different fragments. This method may work well for targeting tens of genes but beyond that scale, it requires more effort to design unique primers and optimize the PCR amplification process to ensure uniformity across all fragments. Also, the high cost of primer design and amplification will not compare favorably with that of sequencing as sequencing costs have been reduced significantly. To overcome the cost and effort of a primer design process, Porreca *et al*. have developed an assay that uses a microarray to design the oligonucleotides in parallel [15]. Using a modification of MIP (molecular inversion probes) assay, 55,000 exons of sizes varying from 60 to 191 bp were targeted and although the specificity of the capture was very good, only ~11,000 exons were captured.

At the same time when Porreca *et al*. published, Albert *et al*. and Okou *et al*. described methods for using DNA arrays to capture large genomic loci directly [15-17]. In the following month, Hodges *et al*. demonstrated success at capturing 'all' exons using the same methods with better balance of coverage (uniformity) and specificity relative to any other previous capture assays, and it was the least labor intensive and the most cost-effective method [18]. However, the performance of these assays varied across the sample types and array types used.

Albert *et al*. targeted a total ~5 Mb of sequences for 660 genes dispersed across the genome [16]. They reported the specificity varied with the same array design from 38% to 76% depending on the samples captured. For their tiling arrays encompassing from 200 Kb to 5 Mb around the *BRCA1 *gene, the fraction of the reads mapped to the intended targets varied from 14% to 64%. The Nimblegen 385 K custom array was used for all capture protocols while the 454 GLX sequencer was used for sequencing. Since the 454 sequencers produce longer individual reads than those from ABI or Illumina sequencers, their sequences are easier to map to the correct genomic location and this should be factored in when comparing capture technologies.

Hodges *et al*. targeted 'all' human exons using seven Nimblegen arrays and sequenced the captured DNA with the Illumina sequencer [18]. They first hybridized 500 bp genomic fragments to all seven arrays, and the fraction of the reads mapped to the targeted regions varied from 36% to 55%. When they extended the definition of the targeted region to include 300 bp upstream and downstream of each exon, the targeting efficiency was increased to 55-85%. Next, they used 100-200 bp fragments to hybridize to one of the arrays in an attempt to tighten the sequenced region around the targets. However, the specificity of the intended targets was reduced three-fold with the exon coverage rate up to 99%. For both studies, no detailed interpretation was described for the specificity variations across different array designs and sample types.

Here, we concentrate on improving the capture specificity using consistent sample and array design. Throughout the experiment, paired genomic DNA of both cancer and normal tissues were used from a cancer patient. We approached with two different methods to specifically block the adapters while generating the genomic library for hybridization and investigated two sequential rounds of hybridization. These changes resulted in improvements in our measured specificity of the targeted genomic DNA from the same sample and the same array design.

## Results

### Baseline capture

Initial data demonstrated a specificity of the intended capture intervals of 35% with exon hit rate of 99% using 100-200 bp genomic fragments and protocols similar to the published results and Agilent custom 244 K oligo arrays. These results were comparable with the Hodges *et al*. data using shorter fragments (29% specificity). With these initial data as baseline, we attempted to improve the capture efficiency by changing the hybridization protocol.

### Modified capture protocol to block adapter-adapter hybridization

The first change we made to the Agilent hybridization protocol was to block the adapters ligated at the end of every genomic amplicon in the hybridization mix. We assumed that possible hybridization between different genomic targets based on adapter-adapter hybridization will lead to the inadvertent and non-specific enrichment of off-target fragments. This is due to the fact that all of the genomic fragments in the hybridization mix are flanked with the same Illumina adapters (52 nt and 34 nt), which have comparable length to the intended genome location specific target probes (45-60 nt). Thus, the melting temperature between the adapter hybridizations will be similar to that between the appropriate genomic fragment and its specific probe. Moreover, the effective concentration in the hybridization of the adapter sequence is approximately 10^7 ^fold higher than the genome specific sequences. Thus, these adapter mediated hybridization may dominate the hybridization process.

We tried two different approaches to overcome this non-specific pull down issue. First, to remove complementary adapter strands from the hybridization mix, we separated the two strands of genomic fragments and used one of the strands for the hybridization. To accomplish this, we biotinylated only one of the PCR primers (primer1.1) used in the generation of the genomic library. After the PCR step, the amplicon was bound to streptavidin beads, and the non-biotinylated strand was collected and hybridized to the array. The second approach we tried was mechanistically easier to prepare than separating the two strands. For this method, we added 10 fold molar excess of Illumina primers to the hybridization mix assuming that the primers will bind to all of the adapters flanking the genomic fragments and block them from hybridizing to other adapter sequences on different genomic fragments. It was shown that both approaches increased the specificity to ~60% with more even coverage resulting from the simpler blocking approach, which is the preferred protocol (Table 1).

**Table 1:** Summary of the results.

	Primers	Single strand separation	Double Hyb	Specificity	Exons with reads*	Bases Covered*
Baseline	-	-	-	35%	98%	75%

Single Strand	-	Yes	-	54%	99%	92%

Primer Block	Yes	-	-	62% (63%^†^)	99% (99%^†^)	88% (94%^†^)

Double Hybridization	Yes	-	Yes	90% (82%^†^)	98% (98%^†^)	84% (89%^†^)

* Not considering flanking regions

† Results from replication using the tumor genomic DNA.

4 μg of genomic library was used for all experiments. The hybridization mix contained 50 μg of Human Cot-1, 52 μl of Agilent 10× Blocking agent and 260 μl of Agilent 2× hybridization buffer. Specificity is calculated by dividing sequence counts within the targeted region by total sequence counts mapable to the human genome uniquely for each run. Targeted region is defined as the targeted exon +/- 100 bp.

### Modified capture protocol with hybridization repeat

The other protocol modification tested was to repeat the hybridization step with the notion that each round of successive hybridization will further enrich for the target sequence as a substantially simplified amplicon is hybridized in the second round. In 2005, Bashiardes *et al*. reported in Nature Methods on capturing genomic loci using bacterial artificial chromosome (BAC) in solution [19]. They performed two rounds of hybridization to enhance targeting and achieved 50% specificity. We incorporated this idea and repeated the hybridization step after the 2^nd ^PCR. The targeted specificity was successfully increased up to 90% (Table 1).

This two step modified protocol was independently replicated externally. Total of 2 Mb was targeted for 7,475 non-overlapping exon intervals under 10 linkage regions across the genome. The capture was done by Agilent custom 244 K oligo arrays strictly following the presented protocols except for reducing the 2^nd ^hybridization time to 24 hrs and re-using the same capture array for the 2^nd ^hybridization. The 36 bp single end sequencing was performed using Illumina GA I in the authors' laboratory and the sequences were aligned to the whole genome using MAQ (Mapping and Assembly with Qualities). With the use of blockers, the specificity resulted in 44% and by repeating the hybridization with the blockers, the specificity increased to 84%, which are comparable to the data presented.

### Analysis of the sequence data

**Figure 1:**
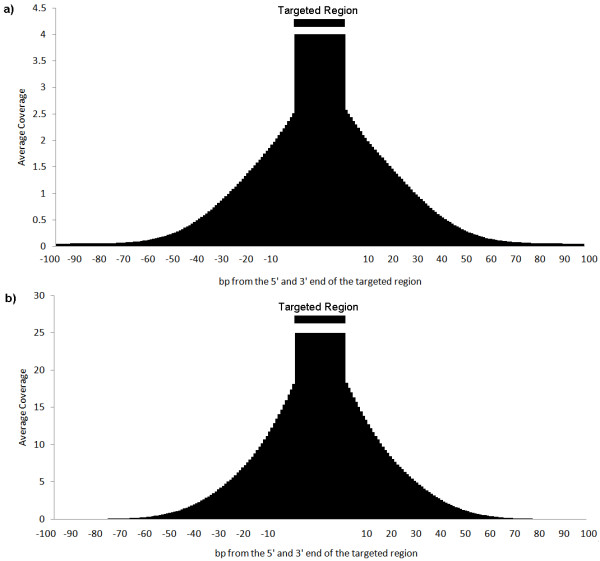
**Mapping of sequences relative to probe position in the genome**. a) Sequence coverage distribution averaged across all targeted regions captured by basal capture protocol and b) sequence coverage distribution averaged across all targeted regions captured by double hybridization (modified) protocol show that the sequence reads are tightly limited around the targeted regions. Here, a targeted region is not necessarily a targeted exon but a probeset composed of multiple probes that are < 200 bp apart to each other. The y axis plots the relative abundance and the x axis is the base position relative to the probes positions.

For both the basal and modified capture protocols, only a few percent of sequences mapped beyond 100 bp upstream or downstream from the end oligo sequence boundaries reflecting the sharpness of the capture which was determined by the fragment library size initially created (Figure 1). It has been shown in the previous reports that SNPs (single nucleotide polymorphisms) can be reliably detected [16-18]. For validation of the variant calls, we compared the captured data to the Illumina 1 M Duo genotyping array data of the same sample. There were 5,746 dbSNP129 SNPs that were present on both the 1 M Duo genotyping array and within the targeted amplicons. The amplicons were sequenced an average of 6× for single hybridization and 9× for double hybridization. 6.3% and 9% of the polymorphic positions were not sequenced in single and double hybridization, respectively. Excluding these positions, the false negative rate (missing the variant allele from capture data while detecting it by the Illumina genotype data) was calculated to be 7.1% and 8.4% for the single and double hybridization, respectively. The false positive rate (detecting the correct variant allele according to the HapMap Caucasian data when Illumina genotype data calls it homozygous reference) was less than 0.1% in both experiments. Although random sampling effect was observed, the range of the variant allele detection ratio at the polymorphic positions was narrowed as the coverage increased for both experiments (Additional file 1). In addition to base substitutions, we have detected small (< 3 bp) insertions and deletions in our dataset that are described in the dbSNP129. Further, novel indels have been discovered and validated from cancer samples that will be described more completely in another publication.

**Figure 2:**
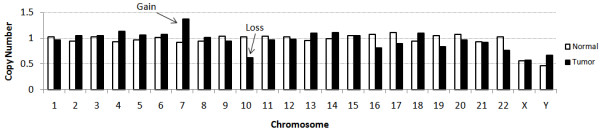
**Copy number fold differences between the normal and tumor tissues per chromosome using single hybridization capture protocol with blockers**. The cancer specimen used in these experiments was known to have a chromosome 7 copy number gain and a chromosome 10 deletion. The normalized counts per chromosome are plotted for all chromosomes and are markedly different for the two chromosomes at altered copy numbers.

**Figure 3:**
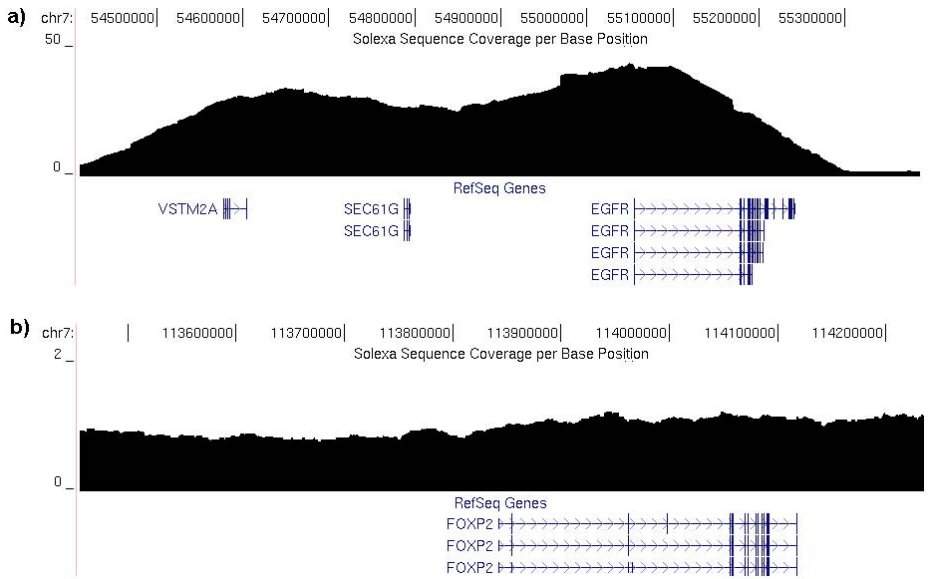
***EGFR *DNA amplification event is preserved in sequence data**. A 200 Kb sized moving average of the interval flanking a) known *EGFR *amplification event are plotted in genomic position and b) for reference another genomic interval around the *FOXP2 *gene also on chromosome 7 is shown demonstrating the more typical coverage. The *EGFR *region is amplified 25× in average compared to the region outside of *EGFR*.

We also gathered information about the frequency of sequence observations and their correlation with copy number in the targeted genome post single hybridization based pull down. Since we used both cancer and normal tissue samples from a single cancer patient, we compared the copy number differences between the two (Figure 2) based on the relative frequency of reads mapping to specific chromosomes. In this experiment, the cancer sample was trisomic for chromosome 7 which had been previously determined by whole genome SNP typing (data not shown). The mean number of counts normalized for the physical length of chromosome in the tumor tissue was 1.4 relative to that in the normal tissue. Further, the cancer sample had a loss of one chromosome, which we observed to have 0.65 the number of reads as the same chromosome in the normal tissue. These results indicate the capture method in aggregate preserves the copy number state of the original genomic DNA, and may be useful for copy number detection even when using the capture method. This is important for the identification of larger deletions using sequencing based approaches. Out of 18 places in the genome that showed regional copy number changes in the cancer sample by whole genome SNP typing, 6 of them harbored captured gene(s) and all except for one place agreed between the two datasets (Table 2). However, considering that there was no SNP placed by Affymetrix 250 K array within the disagreed genic region and the low resolution of the Affymetrix 250 K array for detecting copy number changes, it is likely that the copy number changes detected by the captured sequencing may be true. The sample was also known to have DNA amplification of *EGFR*, "epidermal growth factor receptor", and a focused observation indicated ~25× fold more reads mapping to the *EGFR *exons in tumor sample than in normal sample (normalized to average coverage of all targeted exons across the whole genome) when single hybridization capture protocol was applied to both samples. We note that this is substantially higher than the SNP based copy number data which indicated a 2 fold increase at *EGFR *and may indicate higher dynamic range from the capture approach followed by sequencing than a microarray approach which has a limited dynamic range due to fluorophor measurement. In addition, the background noise for the ~1 Mb flanking region of *EGFR *also showed the ~25× fold amplification compared to elsewhere on the same chromosome reflecting that this 1 Mb region containing the *EGFR *gene is amplified itself at the same ratio (Figure 3).

**Table 2:** Genomic intervals with regional aberration detected by SNP array were examined by capture array and the results were compared.

Genomic Interval	SNP array	Capture array
chr1:141510591-142763403	Gain	None captured

chr1:92652000-93425291	Gain	Gain

chr2:42528749-43214954	Loss	None captured

chr3:52074481-55997844	Gain	None captured

chr6:144171325-144331154	Gain (4 copies)	Gain (25 copies)

chr6:164267664-164772727	Loss	Loss

chr6:169375842-169748900	Loss	None captured

chr6:56774326-56953414	Loss	Loss

chr7:54464822-55373694	Gain	None captured

chr9:14995594-24802191	Gain	None captured

chr14:68301617-68906063	Loss	None captured

chr15:38336909-38504594	Gain	Loss

chr16:2652029-2764985	Loss	None captured

chr17:5541586-5968563	Gain	No Change

chr20:39176664-39815654	Gain	None captured

chr20:61246745-61325513	Gain	None captured

chrX:13343420-151922021	Gain	None captured

chrX:1674881-1838306	Loss	None captured

## Discussion

Both Nimblegen and Agilent have released their commercial products for capture. However, Nimblegen's protocol is specific for the Roche 454 sequencer and no details of the hybridization mix contents are provided. Agilent's protocol uses solution based oligos and although the protocol can be adjusted for either Illumina GA or ABI SOLiD sequencing, it is not cost effective yet for small number of samples. Here, we described a complete instruction of the improved capture protocol with a troubleshooting guide (Table 3) that should facilitate the preparation of enriched genomic libraries given access to either Agilent or Nimblegen hybridization equipment and any of the next generation sequencers and be applicable to other genomes.

**Table 3:** Troubleshooting Guide

Problem	Possible Reason	Solution
Genome is not fragmented after sonication.	Buffer condition is not adaptable for sonication.	Purify the DNA. We used QIAGEN PCR Purification Kit, eluted in EB to have it work.

Nothing is visible on the gel after 1 hr electrophoresis during library generation.	When the starting amount of DNA is small or there is significant DNA loss during the process for various reasons, it is possible that the DNA is smeared over a wide range after an hour of electrophoresis and not visible on the gel.	It is good to check the gel to see if the DNA is present after ~10 min run when the DNA is not smeared at a wide range. Even though nothing is visible, it is highly possible that the DNA is still present. Proceed to the next step regardless and see if PCR amplifies anything.

Cannot collect ~400 ul after the stripping step.	Gasket slide was re-used. The array slide was lifted up too quickly. Different buffer was used for the 95C stripping process.	Do not re-use the gasket slide. The solution can be flushed to a collection boat and collected. When using multi-array slides like 2×105 K, 4×44 K or 8×15 K, it is still possible to run the capture protocol as indicated. After the stripping, the array slide should be slowly lifted up to prevent contamination. The solution tends to stay within the gaskets.

Not enough DNA amplified after the first stripping.	Stripping was not efficient.	Another stripping process can be done and checked if there were left over genomic fragments hybridized on the probes. Since it does not matter if the stripped solution contains contaminants as long as the contaminants do not have adapters ligated at the end, it is possible to thoroughly continue the stripping process until no products get amplified.

Two simple optimizations of the hybridization protocol have improved the capture performance significantly. First, by blocking the adapter sequences flanking each of the genomic fragments, we reduced the non-specific pull down through adapter-adapter hybridization. Blocking the nonspecific DNA is an old trick to reduce the background when microarray experiments are performed, with human cot-1 being the most commonly used reagent to block repetitive sequences [20]. Recently, Hodges et al. has shown similar results with the same approach, validating our experimental protocol [21]. Secondly, we repeated the hybridization step to further enrich the genomic fragment pool. While the specificity was enhanced up to 90%, this step introduced ~1% of variant loss and some degree of bias in the relative abundance of specific amplicons. For example, the fold difference observed for *EGFR *gene was weakened by 2.5-fold when the double hybridization capture protocol was applied, suggesting saturation of the hybridization step effectively normalizing the yield from each amplicon. The overall correlation coefficient between the single hybridization experiment and the double hybridization experiment after excluding the ~100 exons that were outlier was 0.82. This interferes some with the ability to reliable call copy number state of individual exons from the pull down sequence data. Two-round hybridization should be used with caution when copy number detection is critical. The array designed for our current experiments and those in previous reports were all masked for repeats. To test whether including the repeat regions would affect the capture, we have attempted to tile every 15 bp across a 4 Mb region of a single chromosome using Agilent 244 K custom array without vigorously masking for the repeats. The specificity was significantly reduced to 15~30% even with the addition of the primer blockers and increased human cot-1 DNA in the hybridization mix (data not shown). This phenomenon should be taken into account when it is unavoidable to target the repeat regions.

Throughout the experiments, the sequence reads generated were tightly mapped nearby the intended probe regions. For each probe, the local sequence coverage will extend out in relation to the length of genomic fragment library initially created. Without any major variations in the genomic fragments that could interrupt the hybridization to the probe, the sequence coverage will peak within the probe region and decrease with increased distance.

**Figure 4:**
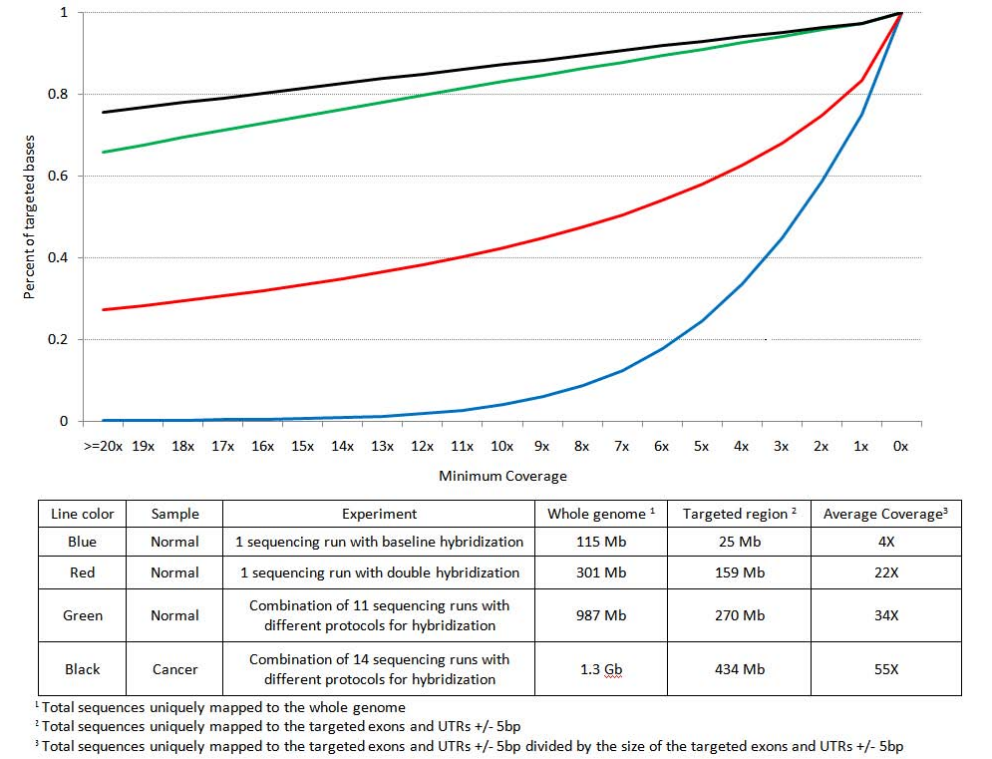
**Percentage of targeted bases sequenced at various minimum coverage for different mean coverages**. X-axis represents the coverage per base level and the corresponding y-axis represents the percentage of targeted bases that were covered at greater or equal with certain coverage. Table legends describe the detail of each line shown.

There are ~18,000 genes in RefSeq database composed of ~33 Mb of coding sequences. To tile every 30 bp, ~914 K probes are needed to be designed which is possible to accomplish on a total set of four Agilent 244 K custom arrays or one Agilent 1 M custom arrays. Figure 4 shows the proportion of 8 million targeted bases covered at various minimum coverage for different mean coverage within the targeted regions. For example, 76% of the targeted bases were considered completely sequenced with sequence depth of 20× or more when the mean coverage within the targeted regions was 55×. From these data, we can project how many sequence reads are required to comprehensively sequence all RefSeq exons. In this report, we used 36 bp of single end sequence reads generated by Illumina GA I. Currently, longer reads of 76 bp paired end sequence reads can be generated and are of sufficient quality for resequencing by Illumina GA IIx. This improvement not only increases the total sequences read by one channel of a flowcell, but also facilitates the alignment to the genome significantly. On average, 2.5 Gb of sequences are generated by one channel of Illumina GA IIx run. Of this, about half of the sequences are mapped uniquely to the human genome and assuming 60-85% specificity of capture, we will be able to generate 0.75-1.06 Gb of sequences within the targeted region. If targeting 33 Mb of the human genome for all RefSeq coding exons, it will require 2 channels (quarter a machine run) of sequencing with Illumina Genome Analyzer (GA) IIx to achieve 20× or more coverage on ~80% of the targeted sequences for one sample: or four samples can be sequenced with each machine run. Alternatively, each run of the ABI SOLiD 3 Plus instrument can generate up to 1 billion 50 base paired end reads, and a total of 40 Gb of mapped genomic sequence, such that 12 exomes can be resequenced at comparable coverage with each machine run (S. Nelson, unpublished results). Thus, whole transcriptome resequencing is economically feasible on the current generation of capture tools and sequencing devices, and, in principle, can be performed for under $2000 per genome.

## Conclusions

Capturing genomic regions for sequencing has a wide scope of application. Many genetic studies with linkage or association signals will benefit immensely as it becomes possible to reliably and inexpensively capture the region of interest and perform high throughput, shotgun sequencing. Additionally, improvements in exonic enrichment protocols will usher in an era of cost effective sequencing of all the amino acid coding bases of genomes. This will lead to more rapid identification of the causative genes in many disorders.

## Methods

### Array Design

We chose to capture exonic sequence of ~3,000 cancer genes. Two cancer gene lists, 'cancer gene census list' and 'CGP (cancer genome project) planned studies list' were retrieved from Wellcome Trust COSMIC (catalogue of somatic mutations in cancer) database and combined[22]. Boundaries for exons and UTRs (untranslated regions) were retrieved from RefSeq database and any intervals that overlapped were merged so that non-redundant contiguous intervals were generated. In total, 3,038 genes (31,678 exons) spanning 8.4 Mb were included in the list. Based on the preliminary capture experience data (not shown), we tiled the probes every 120 bp on average so that the distance between the start positions of the two consecutive probes is ~120 bp and the regions between the probes are covered by the two flanking probes resulting in the same coverage as the regions within the probes. Both forward and reverse strands of each probe region were spotted on the array and 3 of the genes (150 exons) of higher interest were spotted at 12× (6× for each strand). Instead of using Nimblegen arrays as other studies have, we used Agilent 244 K custom 60 mer arrays. Probe design was performed using Agilent e-array system http://www.Agilent.com with the repeat mask function on. This resulted in losing 155 exons completely and ~27% of the exons were partially covered (see Additional file 2).

### Sample Preparation

We used a paired normal and tumor whole genomic DNA as the starting materials. The tumor DNA was extracted from the glioblastoma (GBM) specimen and the normal DNA was extracted from the blood sample of the same patient. The collections were approved by the UCLA IRB and the samples were processed at the Biological Samples Processing Core at UCLA using Autopure LS™ nucleic acid purification instrument from Gentra Systems. Both the normal and tumor samples were run on the Affymetrix GeneChip Human Mapping 250 K array for global genomic examination and comparison. Both chromosomal and regional copy number aberrations were detected in the tumor sample with the hallmarks of glioblastoma like *EGFR *amplification and chromosome 10 loss observed. The detailed description of the mutational landscape and the chromosomal abnormalities of this cancer sample is in preparation (Lee et al.) As we were sequencing with the Illumina Genome Analyzer, we followed the Illumina library generation protocol version 2.3. Five μg of high molecular weight whole genomic DNA from each sample were diluted in 150 μl water as the starting material. The DNA was sheared using a sonicator (Bioruptor, Diagenode) for 1 hr at high power level to generate short fragments. The size of the sheared product ranged between 150 bp to 400 bp with the median size around 200-250 bp. The sample was concentrated in 30 μl of elution buffer (EB) after purification using QIAquick PCR Purification Kit (Qiagen). To repair 3' or 5' overhangs and generate blunt ends for all the double stranded fragments, 5 μl of T4 DNA polymerase, 1 μl of Klenow DNA polymerase, 5 μl of T4 PNK, 4 μl of dNTP mix, 10 μl of T4 DNA ligase buffer with 10 mM ATP and 45 μl water were added to the DNA solution and incubated at 20°C for 30 min and purified to be eluted in 32 μl of EB. Single 'A' base was added to the fragments in a reaction with 3 μl of Klenow exo- (3' to 5' exo minus), 10 μl of dATP and 5 μl of Klenow buffer and incubated at 37°C for 30 min. After purification with MinElute PCR purification kit (QIAGEN) and elution in 10 μl of EB, the Illumina supplied adapters were ligated to the genomic fragments. Five μl of DNA ligase, 10 μl of adapter oligo mix, 25 μl of DNA ligase buffer were used and the sample was incubated for 15 min at room temperature. To select a size-range of templates (100-200 bp) with the correct adapter pairs ligated, the templates were purified and size fractionated on a 2% agarose gel for 1 hr at 120 V. A band from 150-250 bases (including the adaptors) was excised, gel extracted using Gel Extraction Kit (Qiagen) and eluted in 30 μl of EB. The template was PCR amplified with 1 μl of template DNA, 25 μl Phusion DNA polymerase, 1 μl of Illumina primer 1.1, 1 μl of Illumina primer 2.1 and 22 μl of water. Four replicates of PCR reactions were performed to generate sufficient genomic amplicon of ~4 ug for successful hybridization. Total of 18 cycles of PCR with the following cycle conditions was performed: 30 sec at 98°C, [10 sec at 98°C, 30 sec at 65°C, 30 sec at 72°C] × 18 cycles, 5 min at 72°C and hold at 4°C. Samples were purified, eluted in 50 μl of EB and quantified with spectrophotometer (Nanodrop).

### Capture

#### Hybridization

Hybridization was performed according to Agilent CGH 244 K array protocol. 4 μg of amplified products were mixed with 50 μg of Human Cot-1 DNA (Invitrogen, Carlsbad, CA), 52 μl of Agilent 10× Blocking Agent, 260 μl of Agilent 2× Hybridization Buffer (Agilent) and 8 μl of each of the Illumina primer stock (100 μM each) in a final volume of 520 μl. Blockers were designed to be complementary to the Illumina supplied primers. The sequences provided by Illumina are shown in Table 4 (Oligonucleotide sequences^© ^2006 Illumina, Inc. All rights reserved) and the two primers were separately ordered from IDT (Integrated DNA technologies). The hybridization mix was denatured at 95°C for 3 min and pre-hybridized at 37°C for 30 min. 490 μl of the prehybridized amplicons were dispensed to a gasket slide settled in a hybridization chamber base (Agilent), then covered with the array slide and tightly locked with the cover chamber. Hybridization occurred in a rotating oven (Robbins Scientific) for 65 hours at 65°C, 20 rpm.

**Table 4:** PCR primer sequences used to design primers for blocking the adapters in the hybridization mix.

Primer 1.1	5' AATGATACGGCGACCACCGAGATCTACACTCTTTCCCTACACGACGCTCTTCCGATCT
Primer 2.1	5' CAAGCAGAAGACGGCATACGAGCTCTTCCGATCT

Oligonucleotide sequences^© ^2006 Illumina, Inc. All rights reserved.

#### Washing

After hybridization, the arrays were washed according to the Agilent CGH wash procedure A protocol with the 2^nd ^wash extended to 5 minutes for increased stringency. The chamber was disassembled and the array slide was separated from the gasket slide in a glass dish filled with Oligo aCGH wash buffer 1 (Agilent). The array slide was placed on a slide-rack in another glass dish filled with Oligo aCGH wash buffer 1 and washed for 5 min at room temperature with stirring on a magnetic stirring plate. The slide rack was carefully moved to a glass dish filled with Agilent Oligo aCGH wash buffer 2 that was pre-heated to 37°C in a water bath and was washed for 5 min. After washing, the arrays were transferred back to a glass dish with Agilent Oligo aCGH wash buffer 1 until the next step was ready.

#### Stripping

490 μl of 1× Titanium Taq PCR Buffer (Clontech) pre-heated to 95°C was dispensed to a new gasket slide and covered with the array slide that was washed. After securely locking the arrays in the chamber, the array was incubated in the 95°C rotating oven for 10 min at 20 rpm. After this stripping process, the chamber was disassembled and the array slide was carefully lifted up from one side so that the solution converged on the gasket slide. Pipette was used to collect the solution and transfer to a 1.5 mL microtube. This step needed to be done promptly as the solution was heated to 95°C and it started evaporating quickly. Collected solution was approximately 400 μl. The sample was aliquoted into 4 tubes so that when added with the Illumina primer pair (final concentration 0.1 μM), enzyme (final concentration 0.5×), and dNTPs (final concentration 250 μM each), the final volume were 100 μl. The stripped DNA was amplified by 15 cycles of PCR as described previously. The samples were purified with QIAquick PCR purification kit, consolidating and eluting the sample in 50 μl of EB. The concentration of DNA was measured and the size was checked on a 2% agarose gel to confirm that the size matched the size extracted from the gel in the previous step. Hybridization, washing and stripping steps were repeated. The stripped DNA was amplified under the same condition as before. After checking the concentration and template size again, the sample was diluted in a final concentration of 10 nM, which is the working concentration for cluster generation.

### Sequencing

The Illumina flowcell was strictly prepared according to the manufacturer's protocol and the clusters were sequenced on the Genome Analyzer using standard manufacturer's recommended protocols. The image data produced were converted to intensity files and the sequential image data were processed through the "Firecrest" and "Bustard" algorithms provided by Illumina were used to call the individual sequence reads.

### Alignment

We used the Blat-like Fast Accurate Search Tool (BFAST, in submission) to map each sequence read back to its location in the reference genome. Here we used the NCBI human genome build 36 as our reference genome [23]. Ten different genome indexes (Table 5) were built for use in the BFAST alignment process to be robust to errors and variants in the short (typically 36 base pairs) reads used throughout this project. For each read, the potential genome locations identified by BFAST were evaluated using a standard local alignment algorithm and ranked by score (see http://genome.ucla.edu/bfast). The best scoring alignment for each read was chosen, while reads with multiple top-scoring alignments or no alignments were discarded. MAQ was run on the same dataset to ensure we do not see any bias introduced by the alignment program. The aligned reads were post filtered so that the reads with mapping quality 0 that are aligned to multiple places in the genome were removed. The difference of the number of reads uniquely mapped to the whole genome (paired t-test p-value: 0.14) and the specificities for each experiment (paired t-test p-value: 0.13) were not significant between the BFAST aligned data and MAQ aligned data (Additional file 3). Two summary reports were created once the most likely alignment for each read was identified by BFAST. The first report was a BED file describing mismatches between the Illumina sequence and their corresponding genomic sequence. The second report was a Wiggle (WIG) file describing sequence coverage at each position along the chromosomes. These file formats were used because they are compatible with the popular UCSC genome browser. The open source SeqWare project (in submission), which provides a LIMS tool for tracking samples (SeqWare LIMS) and a pipeline for sequence analysis (SeqWare Pipeline), http://seqware.sourceforge.net was used throughout this work. It streamlined the sequence processing by running the Illumina-provided image analysis and base-calling tools, BFAST, and the report generation code, which itself is part of the SeqWare project. The BED file was further mapped to the dbSNP129 database to filter out known SNPs from *de novo *variants.

**Table 5:** Masks that define each index for sequence alignment.

Index	Masks
1	111111111111111111

2	11110100110111101010101111

3	11111111111111001111

4	1111011101100101001111111

5	11110111000101010000010101110111

6	1011001101011110100110010010111

7	1110110010100001000101100111001111

8	1111011111111111111

9	11011111100010110111101101

10	111010001110001110100011011111

### Data Analysis

We counted the sequences that were mapped back to the targeted region to calculate the specificity. From the WIG file for each chromosome, the base positions that mapped within the target intervals were filtered, and the sequence counts for each base position were summed. The target intervals were defined as extending 100 bp upstream and downstream from the final oligo intended to capture each exon interval. To compare the copy number of each chromosome between the normal and tumor sample from a patient, sequence counts were first tallied for each target interval and divided by total sequence counts within all target intervals for normalization. Mean of sequence counts per chromosome was calculated and compared between the normal and tumor sample. To compare the background noise between flanking region of *EGFR *and *FOXP2 *genes on chromosome 7, only the coverage in the non-targeted region (excluding targeted region +/- 100 bp) was considered. 200 Kb moving average of the coverage was calculated across each region.

## Authors' contributions

HL and BM conceived and designed the experiments. HL performed the experiments and wrote the paper. HL and BO analyzed the data. VAF and DHC contributed the replication dataset. NH contributed the analysis tools. ZC contributed the reagents and supervised the sequencing run. SN supervised the project as PI on the grant. All authors read and approved the final manuscript.

## Supplementary Material

Additional file 1 **The variant allele ratios for each polymorphic position that were called by Illumina 1 M duo genotype data**. The x-axis is the coverage per base and the y-axis is the variant allele ratio. Each spot is a polymorphic position that the capture data intersected with the Illumina genotype data. Different colors represent either homozygous reference (blue), homozygous non-reference (red) or heterozygous (black). Click here for file

Additional file 2 **Oligonucleotide Capture Probes**. The table lists the sequence and the chromosomal locations of the oligonucleotide capture probes used. Click here for file

Additional file 3 **Summary table**. Summary Table of the Illumina Runs for both BFAST and MAQ alignment. Click here for file
