# Supplementary figures and images for: Improving the efficiency of genomic loci capture using oligonucleotide arrays for high throughput resequencing
Source: BMC Genomics. 2009 Dec 31;10:646. doi: 10.1186/1471-2164-10-646 (PMC2808330; doi:10.1186/1471-2164-10-646)

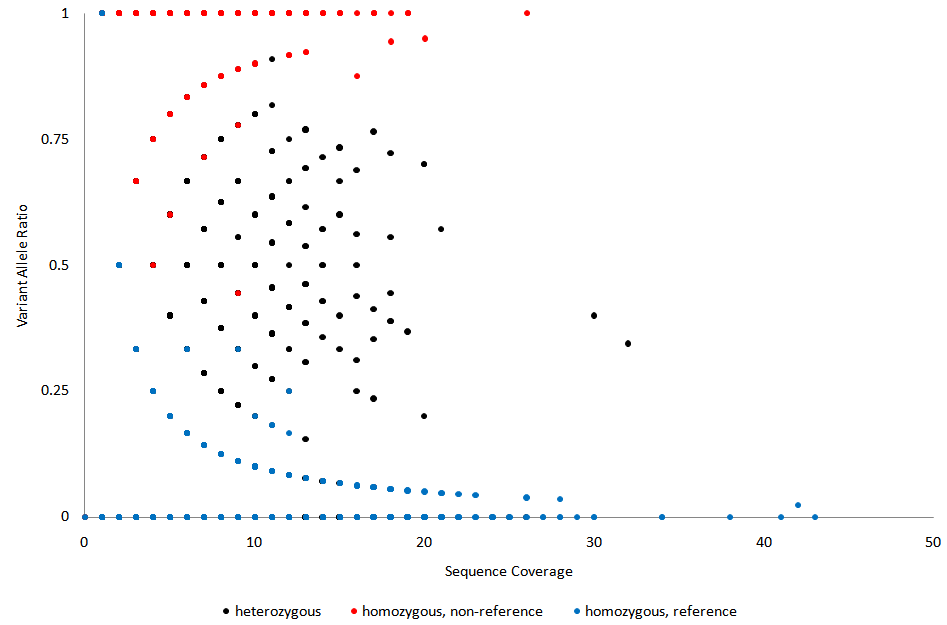

Supplement: Additional file 1 — The variant allele ratios for each polymorphic position that were called by Illumina 1 M duo genotype data. The x-axis is the coverage per base and the y-axis is the variant allele ratio. Each spot is a polymorphic position that the capture data intersected with the Illumina genotype data. Different colors represent either homozygous reference (blue), homozygous non-reference (red) or heterozygous (black). [file 1471-2164-10-646-S1.BMP]
