# Supplementary material for: Improving the efficiency of genomic loci capture using oligonucleotide arrays for high throughput resequencing
Source: BMC Genomics. 2009 Dec 31;10:646. doi: 10.1186/1471-2164-10-646 (PMC2808330; doi:10.1186/1471-2164-10-646)
Supplement: Additional file 3 — Summary table. Summary Table of the Illumina Runs for both BFAST and MAQ alignment. [file 1471-2164-10-646-S3.RTF]

BFAST
Index	Library	Hybridization Protocol	Total Reads Generated by Illumina GA	Reads Mapped Uniquely to the Genome	Proportion of Total Reads Mapped to Selection Targets	Mean Fold Coverage	
1	Normal	Baseline	6,653,871	3,838,977	35%	4X	
2	Normal	Primers Added (PA)	5,446,838	3,995,321	62%	8X	
3	Normal	Single Strand hybridization	7,538,318	4,513,811	54%	7X	
4	Normal	PA + Double hybridization	13,967,432	9,060,688	90%	22X	
5	Tumor	Primers Added	6,283,247	4,093,758	63%	7X	
6	Tumor	PA + Double hybridization	7,150,470	4,841,095	82%	11X	

MAQ
Index	Library	Hybridization Protocol	Total Reads Generated by Illumina GA	Reads Mapped Uniquely to the Genome	Proportion of Total Reads Mapped to Selection Targets	Mean Fold Coverage	
1	Normal	Baseline	6,653,871	3,994,604	33%	4X	
2	Normal	Primers Added	5,446,838	4,021,395	66%	8X	
3	Normal	Single Strand hybridization	7,538,318	3,912,470	55%	6X	
4	Normal	PA + Double hybridization	13,967,432	7,342,127	92%	24X	
5	Tumor	Primers Added	6,283,247	3,777,118	64%	7X	
6	Tumor	PA + Double hybridization	7,150,470	4,388,266	86%	11X	
